# Species richness of Orthoptera declines with elevation while elevational range of individual species peaks at mid elevation

**DOI:** 10.1002/ece3.10985

**Published:** 2024-02-21

**Authors:** Jen Thomas, Simon T. Segar, Andrew J. Cherrill

**Affiliations:** ^1^ Department of Agriculture and Environment Harper Adams University Newport UK

**Keywords:** elevational gradient, elevational range, Orthoptera, Rapoport's Rule, species richness

## Abstract

Species richness has been shown to decrease, and elevational range increase (the Rapoport effect), with elevation as a consequence of biotic and abiotic factors, but patterns are inconsistent across taxonomic groups. Despite being an important indicator taxon and a component of local communities, Orthoptera distributions at higher elevations in Europe remain unclear. We investigated the relationship of Orthoptera species richness and elevational range with elevation in the Pyrenees mountains, Europe. We conducted sweepnetting surveys supplemented by hand‐sampling, at 28 sites stratified by elevation, across three study areas. Using generalised linear models, we found that species richness declined with elevation. Elevation was an important predictor of species richness, but sampling effort and vegetation structure (height and cover) also contributed to estimates of species richness. Using a nonlinear regression to model the elevational range of species over the elevational gradient, we did not observe a Rapoport effect, with elevational range peaking at mid‐elevation instead. Smaller elevational ranges of species found at high elevations may be due to a combination of sampling over a restricted elevational range and the presence of specialist high‐elevation species. We argue that our findings are useful for understanding species distributions with elevation at the interface between local and regional scales. Clarifying the biotic and abiotic predictors of species distribution is important for informing conservation efforts and predicting consequences of climate change.

## Graphical abstract

Understanding the distribution of organisms along elevational gradients across a range of latitudes and geographical areas is important to be able to inform conservation measures. Using Orthoptera in the Pyrenees as a study system, we found a decline in species richness with increasing elevation, but no evidence for Rapoport's elevational rule.

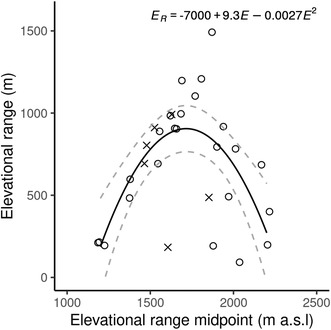

## 1 INTRODUCTION

Elevational gradients represent natural laboratories for exploring patterns of species distribution and diversity. A large body of work across many gradients has demonstrated some generalities but also highlighted that the prevailing distributions largely depend on geographic region, local conditions, taxa and sample design (Almeida‐Neto et al., 2006; Campos‐Cerqueira et al., 2017; Chatzaki et al., 2005; Fleishman et al., 2000; Rahbek, 1995, 2005; Rowe & Lidgard, 2009). As a result of climate change, some species have shifted towards higher elevations (Chen et al., 2011; Lenoir et al., 2008; McCain & Garfinkel, 2021; Wilson et al., 2005) such that some species can find refuge, while others may breach the limits of their climactic niche (Chinn & Chinn, 2020; Lawler et al., 2009; Saraiva et al., 2021). With added pressure from human activities on mountainous areas, understanding species distributions along elevational gradients is crucial for effective conservation and ecosystem management (Saraiva et al., 2021; Thomas et al., 2012; Wessely et al., 2017).

Species richness tends to be lower at higher elevations (Chatzaki et al., 2005; Senyuz et al., 2019) but the exact shape of the species richness‐elevation relationship varies. Rahbek (2005) found that in around 50% of studies species richness peaked at mid‐elevations, and in around 25% it decreased monotonically with elevation. Stevens (1989) proposed that species living at higher latitudes inhabit a greater latitudinal range than those that live at lower latitudes (Rapoport's rule) and in 1992 postulated a similar relationship for elevation gradients. Specifically, Rapoport's elevational rule, hereafter referred to as the Rapoport effect (Colwell & Hurtt, 1994), proposed that there is a positive correlation between the elevational range inhabited by a species and the mean elevation at which it occurs (Stevens, 1992). Stevens (1992) hypothesised that natural selection at higher elevations favours species which tolerate wider climatic conditions, and therefore these species are also able to inhabit a wider elevational range (the Rapoport effect). Stevens (1992) then predicted that species richness is higher at lower elevations because populations of less tolerant species occurring in marginal (sink) low‐elevation habitats are sustained by individuals moving down from higher elevations (Rapoport's rescue hypothesis; Brown & Kodric‐Brown, 1977; Stevens, 1992).

While some studies have reported results in line with the Rapoport effect (Beketov, 2009; Bernadou et al., 2015; Sanders, 2002), this rule is not universal (Bhattarai & Vetaas, 2006; McCain, 2004; McCain & Bracy Knight, 2013; Shimabukuro & Trivinho‐Strixino, 2021). Evidence of the Rapoport effect in insect taxa is inconclusive. Primarily, these studies question the geographic and temporal scale of sampling (Almeida‐Neto et al., 2006; Macek et al., 2021), whether the rule is pervasive across different geographic regions (Gaston & Chown, 1999a), and if the proposed underlying mechanisms affecting species richness are appropriate (Almeida‐Neto et al., 2006; Grytnes & Vetaas, 2002; McCain, 2004; Shimabukuro & Trivinho‐Strixino, 2021). One of the drivers (underlying mechanisms) of changes in species richness with elevation and the Rapoport effect in insects may be decreases in temperature with elevation. However, as ectotherms, many insects regulate their temperature behaviourally, such as by varying the alignment of their bodies with incident solar radiation, and by positioning themselves within microhabitats which act as a buffer to extremes (Anderson et al., 1979; Chappell, 1983). Microhabitats are created by small‐scale habitat features such as rocks, open ground and vegetation, as well as the steepness of a slope and the direction in which it faces (aspect; Nadal‐Romero et al., 2014; Påhlsson, 1974). These environmental factors, therefore, need to be quantified in studies investigating elevational patterns in insect distribution, particularly for taxa such as the Orthoptera, which are known to actively thermoregulate. Orthoptera use these microhabitats to position themselves in sunlight, shade or away from the ground to thermoregulate (Anderson et al., 1979; Chappell, 1983; O'Neill & Rolston, 2007). Indeed, grasshoppers at higher elevations have been found to be more mobile and bask more than those at lower elevations (Samietz et al., 2005). Orthoptera are important indicator species of the environment and are sensitive to changes in habitat land‐use and climate (Cannon, 1998; Cherrill, 2010, 2015), but have not been studied extensively along elevational gradients.

In this study we aim to understand the patterns of Orthoptera species richness and elevational range in the Pyrenees. Assuming that elevation is a significant factor affecting Orthoptera species distribution, we predict that species richness will decrease with elevation, and the elevational range over which species occur will increase with elevation (Rapoport effect). To test these hypotheses, we conducted surveys of Orthoptera at sites along an elevational gradient and used linear models to investigate the relationships between species distribution and elevation.

## 2 MATERIALS AND METHODS

### 2.1 Study location

This study was undertaken in the Alt Pirineu Natural Park (PNAP) in the Catalan Pyrenees, bordering France and Andorra. The Pyrenees mountain range runs from the Cantabrian Sea in the west to the Mediterranean Sea in the east. Elevations within the PNAP range from 650 to 3143 m a.s.l, with several peaks over 3000 m a.s.l. (ICGC, 2022).

A continental climate with cold winters and dry summers is typical in the lower parts of the region (annual temperatures at 990 m a.s.l. range from −11 to 37.7°C), where accessible land has been cleared for agriculture. Sites at these elevations were generally flower‐rich meadows cultivated for pasture, and on some south‐facing slopes, typical Mediterranean oak woodland with clearings for grazing. Mosaic habitats of mature woodlands interspersed with subalpine, flower‐rich grasslands and scrub were found at mid‐elevations where the length of the growing season is balanced between higher rainfall and lower temperatures (Loidi, 2017). The growing season is shortened further at elevations above ca. 1800 m a.s.l. where temperatures can be cold throughout the year (annual temperatures at 1900 m a.s.l. range from −14.5 to 27.8°C). North‐facing slopes generally experience lower temperatures, and in some cases, snow lingers throughout the summer; south‐facing slopes tend to be drier (Loidi, 2017). The tree line gives way to short alpine grasslands at around 2200–2400 m a.s.l (annual temperatures at 2400 m a.s.l. range from −15.9 to 23.3°C). A summary of the region's flora can be found in Loidi (2017).

**FIGURE 1:**
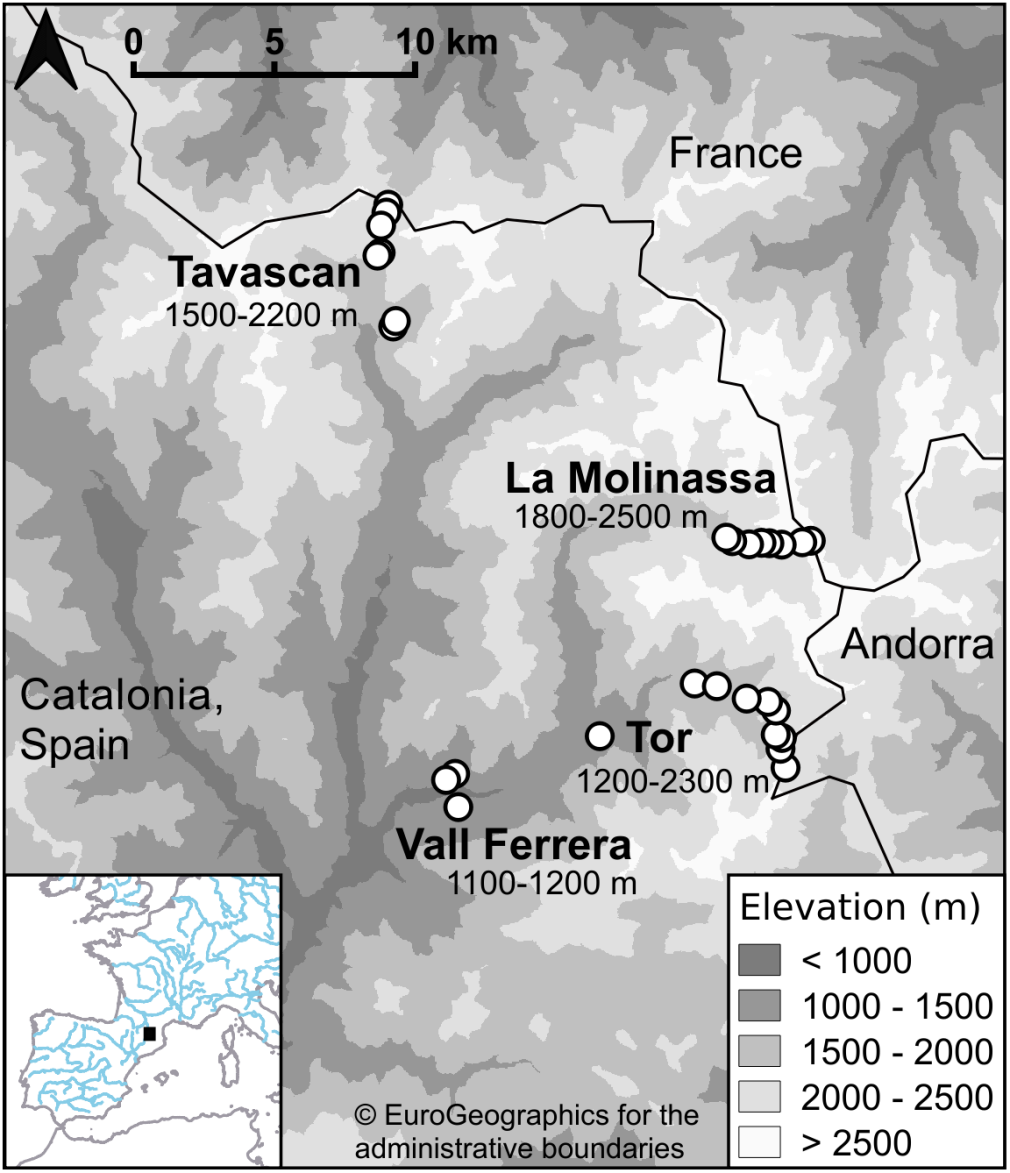
Map of the study areas in the Pyrenees, Catalonia, Spain. The location in the large‐scale map is depicted by the black square in the inset map. The inset map shows coastlines (grey) and main rivers (blue) in western Europe. The white circles represent study sites which are situated within the study areas, identified by the place names in bold font: Tavascan (TAV), La Molinassa (MOL), Tor (TOR) and Vall Ferrera (VFE). Elevational ranges surveyed within each study area are given below the area name. Despite the wider elevation range found in the PNAP, elevations within the study areas ranged from 1000‐2900 m a.s.l and surveys took place over 1100–2500 m a.s.l (valley bottoms at 1000 m a.s.l and higher elevations were not accessible). See Thomas et al. (2023) for elevations of each site. Elevation bands are shown from darker shades representing <1000 m a.s.l. to lighter shades representing >2500 m a.s.l, in 500‐m intervals. The black line delineates the countries which are identified by the non‐bold text. Study sites were chosen using Bing aerial maps (Microsoft Corporation, 2021), digital elevation models (ICGC, 2022) and Open Street Map data (Open Street Map contributors, 2021) using Viking ver. 1.7 (Battaglia & Viking's contributors, 2021). Sources: European hydrography (Efraín Maps, 2020); coastlines (GISCO, 2020a) and administrative boundaries (GISCO, 2020b); background elevation based on data from EU, Copernicus Land Monitoring Service and European Environment Agency (EEA) (2022). Map created using QGIS ver. 3.18. (QGIS Development Team, 2021).

Three study areas, La Molinassa, Tavascan and Tor (Figure 1), were chosen within the PNAP. Within each study area, study sites were chosen within each 100‐m elevational band to give a vertical resolution of 100 m along the elevational gradient. Study sites were chosen to balance their safe accessibility within the required elevational band in the time available and the presence of some open habitat suitable for Orthoptera (i.e. not full tree cover).

All study areas are grazed by horses *Equus ferus caballus* and cattle *Bos taurus* to a varying extent during the summer months, although this grazing appears to be more extensive and intensive at Tor (JT, personal observation), where foot and vehicular access is possible along a rough track. At sites <1800 m a.s.l., vegetation is long and dense during the summer, comprising many flowering species. These sites were generally in smaller open areas surrounded by trees and shrubs and are grazed intermittently. At higher altitudes, sites gradually became more open and were among scattered trees and shrubs of *Pinus uncinata, Juniperus* sp., *Genisteae* sp. and *Vaccinium* sp., among others. Above the treeline, grass and flowering species are short, and other species occur sparsely. Habitat is more semi‐natural at La Molinassa and Tavascan. Access to these areas is only possible on foot, and trails are not well‐defined above 2000 m a.s.l. At higher altitudes above the treeline, vegetation cover is low and short in the case of grassland species, with a large proportion of rock cover above 2000 m a.s.l., particularly at Tavascan. Mountain streams were within 100 m horizontal distance of all sites at la Molinassa except for that at 2500 m a.s.l., and at Tavascan except for those at 1600 and 2200 m a.s.l. Lower elevation sites were in locations with increasing vegetation of species typical of the region (Loidi, 2017).

Across the three main study areas, we surveyed an elevational range of 1100–2500 m a.s.l across 25 sites (Thomas et al., 2023). Three additional sites, located further down the Vall Ferrera (valley) which joins the survey areas at La Molinassa and Tor, were surveyed to provide data at lower elevations (1100–1200 m a.s.l; Figure 1). The elevations surveyed at Tor were 1200–2300 m a.s.l. (10 sites), at La Molinassa were 1800–2500 m a.s.l. (eight sites) and at Tavascan were 1500–2200 m a.s.l. (seven sites; Figure 1). We assigned each study site to an elevational band, which will be referred to throughout this article by its lower value, e.g. a site at 1650 m a.s.l. was in the band 1600–1700 m a.s.l., and is referred to by the elevational band of 1600 m a.s.l. Sites were separated by a horizontal distance of at least 100 m.

### 2.2 Orthoptera surveys

Each site was visited by JT at least twice in summer (June–July) and twice in the autumn (September–October) of 2021, with the exception of four sites which were visited only once in one of the seasons (Vall Ferrera 1100 m a.s.l. and Tor 1200 m a.s.l. once in spring; Tavascan 2100 and 2200 m a.s.l. once in autumn) due to poor weather conditions. In total, 118 site visits were made, with all surveys taking place between 09:00 and 18:00 local time when there was no precipitation.

During a visit, each 100 m‐transect was walked twice at a steady pace to sample for Orthoptera. The first time, a sweep net (opening 0.1 m^2^) was moved at a steady pace through an arc of 180°, reaching as close to the ground as possible. The transect was then walked again, capturing by hand any Orthoptera missed by the sweep net. At sites above 2000 m a.s.l. where conditions were often windy and the vegetation short, another sweep net survey was done to increase the chances of capturing all individuals along a transect. The same transects were walked on each visit.

Due to poor weather, including snow cover at higher elevations, sampling effort varied across sites (number of hand surveys per site: min = 2, max = 3; number of net surveys per site: min = 3, max = 8). To account for uneven sampling effort, and the use of both hand and net sampling, we calculated an index of sampling effort (SE) for each site, which was used in the rest of the analysis as a confounding factor. For each site, we calculated the number of sweepnetting surveys (surveys_NET_), the mean number of specimens caught in a net transect sample (mean_obs_NET_) and the total number of specimens caught in hand‐caught transect samples (total_obs_HAND_). We calculated *SE* for each site using the equation, SE = surveys_NET_ + (total_obs_HAND_/mean_obs_NET_). The second term in this equation converts the number of hand transect samples to the equivalent number of net transect samples that would have caught the same number of specimens.

### 2.3 Species identification

Where possible, we identified Orthoptera to species level in the field or alternatively retained specimens for later identification. Both nymphs and adults were identified using external morphology to the lowest taxonomic level possible using the available keys (Llucià Pomares, 2002; Poniatowski et al., 2012; Sardet et al., 2021). Later‐stage nymphs were identified to species using these keys, where their identification could not be confused.

We calculated species richness for each site by counting the number of unique species observed at a site. Higher taxa were also considered a species for these purposes, where no other species in that taxa were found at a site, or if they were clearly distinct from other taxa within the group (according to where keys diverged). Taxa lists of Orthoptera from all visits to a site were pooled to create a single list for each site.

### 2.4 Environmental variables

We characterised vegetation structure along each transect twice, once in summer and once in the autumn, by randomly selecting three, non‐overlapping plots of 1.2 x 1.2 m. The maximum height of vegetation within the plot was measured, and the height of 75% of the vegetation estimated as in Wettstein and Schmid (1999). Vegetation density was estimated by counting the number of times vegetation touched a vertical rod (diameter 8 mm) placed at the mid‐points of each plot edge, and in the plot centre. Vegetation density from each of the plots was calculated as the average of these five counts. The percentage of ground covered by vegetation, rocks and bare ground within each plot was estimated by eye (Munyai & Foord, 2012). Each measure of vegetation structure was averaged to give one value for each parameter, for each site. Wettstein and Schmid (1999) created an index using the product of these proxy measures of vegetation structure, but we did not combine them, because first, they may affect different species in different ways, and second, they may be correlated with other parameters, such as elevation. All measurements were made by the same observer (JT) to minimise variation.

Slope and aspect were calculated across the study areas using digital elevation models with a resolution of 2 × 2 m (ICGC, 2022). Using the rgeos ver. 0.5–9 (Bivand & Rundel, 2021) and terra ver. 1.5–34 (Hijmans, 2021) packages in R, slope (degrees) and aspect (degrees) were averaged from the four nearest raster cells, every 2 m along the transect. We averaged these across the whole transect, giving one value of slope and aspect for each study site.

### 2.5 Statistical analysis

#### 2.5.1 Patterns of species richness

The species richness in each elevation band was the total number of unique taxa observed within that band. We tested elevation, study area, sampling effort and measures of topography (slope and aspect) and vegetation structure (vegetation cover, average height, maximum height and density) for independence using Spearman's rank correlation. We chose vegetation cover to represent the ground coverage parameter because its values are intrinsically linked to the percentage of ground covered by rocks and bare ground (as the percentage of one increases, the percentage of the others must decrease). Separately, we used Spearman's rank correlation to test the relationship of species richness with (i) elevation, and (ii) sampling effort.

We used generalised linear models (GLMs) with a quasipoisson error distribution (to avoid problems of overdispersion) and a log link function to model species richness (count data) across all sites (Wedderburn, 1974). The full model was constructed using the fixed effects of elevation, study area, sampling effort, vegetation cover, maximum vegetation height, vegetation density, slope and aspect. We reduced the model using backwards stepwise selection. Parameters were removed if they had non‐significant *p*‐values (*α* = .05) and with the aim of minimising the deviance. Analysis of variance (ANOVA) was used after each step to compare the reduced model with the previous step's model, using *F*‐tests.

#### 2.5.2 Rapoport effect

Species are likely to have distinct ecological requirements from others within the same taxonomic group (e.g. *Chorthippus* sp.; Dvořák et al., 2022; Sardet et al., 2021). This could cause incorrect interpretation of results if higher‐level taxa were included in analyses. To test the Rapoport effect, therefore, we used only specimens identified to species (*n* = 616). Species which were only recorded once (singletons) were removed from the analysis (*n* = 7).

Four methods are commonly used to test for the Rapoport effect (Letcher & Harvey, 1994; Pagel et al., 1991; Rohde et al., 1993; Stevens, 1989). Stevens' method considers all species which occur in each elevational band, to estimate the relationship between elevation and mean elevational range (Stevens, 1989), thereby leading to a lack of independence between data points. Instead, Pagel's method uses the most extreme point of the elevational range as a measure of elevation (Pagel et al., 1991). Both Rohde's and Pagel's methods suffer from problems associated with sampling along the boundary of potential ranges (mountain peaks in the case of elevation) and the natural shrinking of available area with increasing elevation (Letcher & Harvey, 1994; Lyons & Willig, 1997). Interdependence between phylogenetically related species (Ruggiero & Werenkraut, 2007) is taken into account in the cross‐species method (Letcher & Harvey, 1994) but this may not affect elevational range size (Blackburn & Gaston, 1998).

Here, we used a modification of Rohde's method (Rohde et al., 1993) as described in Diniz‐Filho and Tôrres (2002), which uses the midpoint of the elevational range as a measure of elevation rather than the mean, to avoid bias created by large numbers of observations at one end of the elevational range (Rohde et al., 1993). The elevational range of each species was calculated by subtracting the lower bound of the lower elevational band in which it was found, from the upper bound of the upper elevational band (Almeida‐Neto et al., 2006; Sanders, 2002). Species which were only observed in one elevational band were assigned an elevational range of 100 m as in Stevens (1992). We calculated the midpoint as being halfway between the minimum and maximum points of the elevational range. Nonlinear regression was used to model the relationship between the midpoint and elevational range of Orthoptera. Regressions up to and including fourth‐order polynomials were fitted. The best model was selected by minimising Akaike's Information Criteria (AIC) and maximising the adjusted *R* ^2^. ANOVA was used to compare the models to each other.

Analyses were conducted in r ver. 4.2.2 (R Core Team, 2022).

## 3 RESULTS

### 3.1 Species assemblages and site conditions

We recorded a total of 1606 individual Orthoptera from 28 sites, comprising 589 adults and 1003 nymphs (with 14 not determined to stage) of which 1418 were Caelifera and 188 Ensifera. In total, 39 taxa, of which 30 were Caelifera and nine Ensifera, were recorded. Of these, 616 individuals (564 Caelifera of 29 species and 52 Ensifera of eight species) were recorded to species level (576 adults, 40 nymphs), giving 37 named species in total. These make up 39% of the Caelifera and 15% of the Ensifera species observed in the Pyrenees above 700 m a.s.l. (Poniatowski et al., 2012). We recorded two species endemic to the Pyrenees, *Cophopodisma pyrenea* and *Gomphoceridius brevipennis*, and a third, *Omocestus antigai*, endemic to the Pyrenees and Catalan range (Poniatowski et al., 2012). Seven species were each represented by a single specimen. Results are based on observations across all visits.

The two measures of vegetation height were highly correlated (*r*_S_ = .64, *p* < .01), as were vegetation density and height of 75% of the vegetation (*r*_S_ = .64, *p* < .01). We chose maximum vegetation height, vegetation cover and vegetation density as measures of vegetation structure to avoid multicollinearity. Although vegetation cover and slope were also highly negatively correlated (*r*_S_ = −.75, *p* < .01), they were each considered important parameters and were both retained in models. Sampling effort was positively correlated with elevation (*r*_S_ = .626, *R* ^2^ = .38, *p* < .001).

### 3.2 Patterns of species richness

**FIGURE 2:**
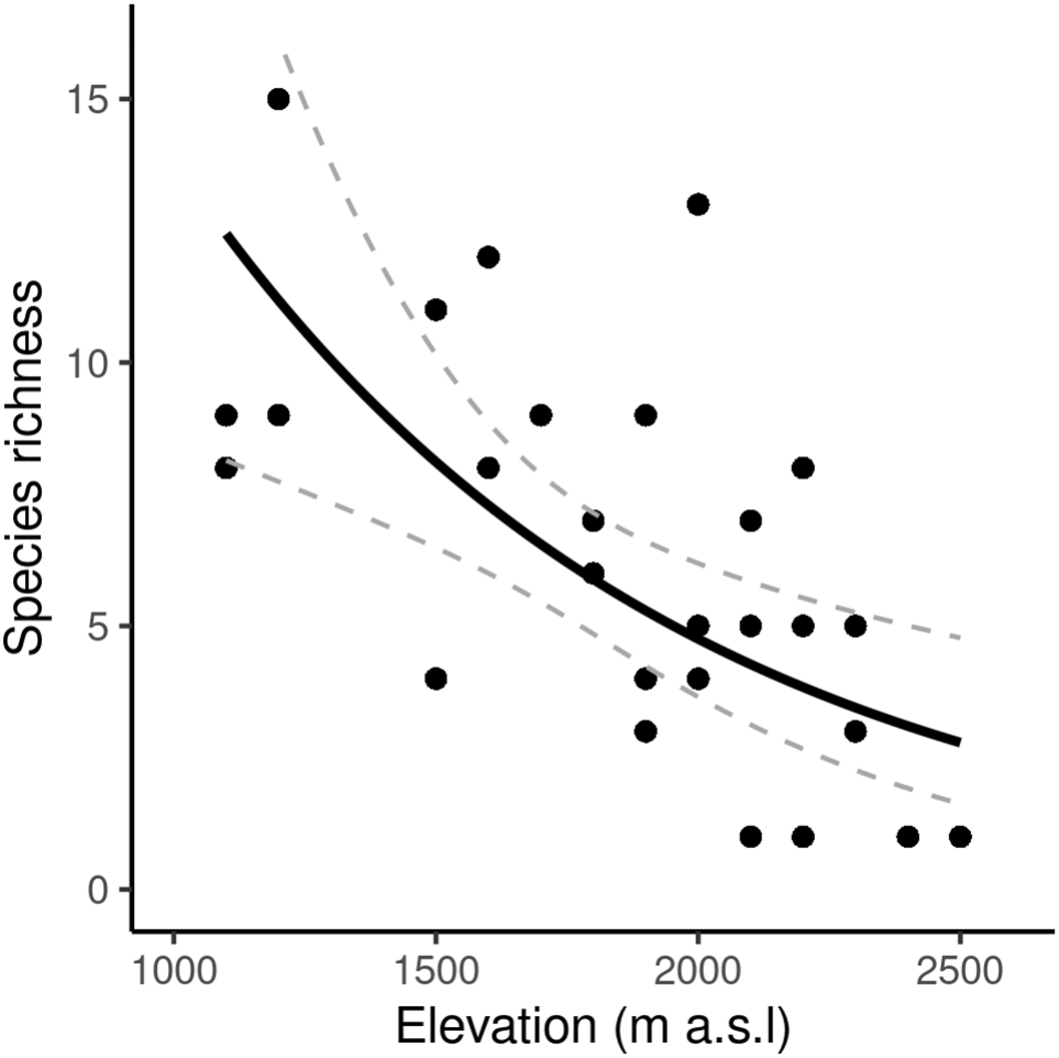
Relationship between Orthoptera species richness and elevation in the Pyrenees. Filled circles are the observed species richness within each elevation band. The solid line represents the fitted GLM (Table 1) and the 95% confidence intervals are bounded by the dashed lines.

The maximum number of taxa observed at one site (1200 m a.s.l. at Vall Ferrera) was 15, with 16 taxa recorded across the 1200 m‐elevation band as a whole. Within the three main study areas, the highest number of taxa recorded at one site was 13 (2000 m a.s.l. at La Molinassa). Only one species was detected at the highest elevations of 2400–2500 m a.s.l (Figure 2). There was evidence to suggest a significant negative relationship between species richness and elevation (*r*_S_ = −.66, *p* < .001); however, only 44% of the variation in species richness was explained by elevation. Sampling effort was not correlated with species richness (*r*_S_ = −.20, *R* ^2^ = .04, *p* > .05).

Species richness was modelled with a GLM using a quasipoisson error distribution and log link function (Figure 2). Elevation, sampling effort, vegetation cover and maximum vegetation height were the most important predictors of species richness after using backwards stepwise selection to remove variables which did not improve the deviance of the model (Table 1). Parameter estimates were similar in the full and reduced models, suggesting that model selection resulted in a satisfactory model.

**TABLE 1:** Generalised linear model for the relationship of Orthoptera species richness with elevation, sampling effort and vegetation structure (cover and height) in the Pyrenees, Europe.

	Parameter estimate (±SE)	*F*	df	*p*
Null deviance = 62.53 (df = 27), residual deviance = 30.32 (df = 23)				
Intercept	2.327 (±0.911)			
Elevation band (m)	−0.001 (±0.0003)	10.71	1	.003
Sampling effort	0.065 (±0.034)	3.49	1	.074
Vegetation cover	0.006 (±0.007)	0.80	1	.381
Maximum vegetation height (cm)	0.011 (±0.006)	3.08	1	.093

*Note*: Number of sites in analysis = 28.

### 3.3 Rapoport effect

**FIGURE 3:**
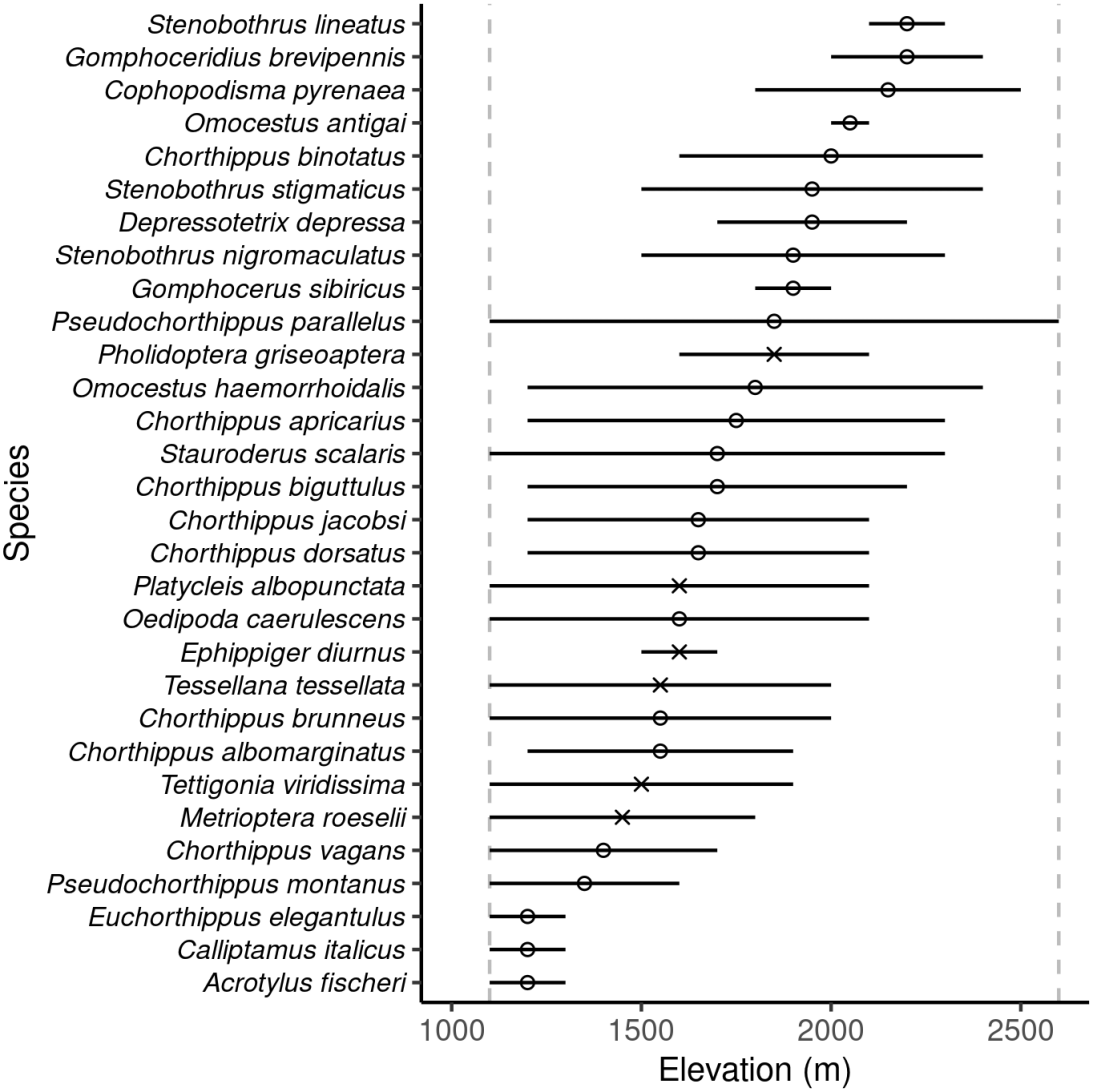
Elevational range of each Orthoptera species in the Pyrenees, ordered by midpoint elevation from highest to lowest (top to bottom). The elevational range over which each species was observed is represented by the solid horizontal lines. The midpoint of the elevational range is represented by a circle (Caelifera) or cross (Ensifera). Vertical dashed lines show the upper and lower elevations surveyed.

**FIGURE 4:**
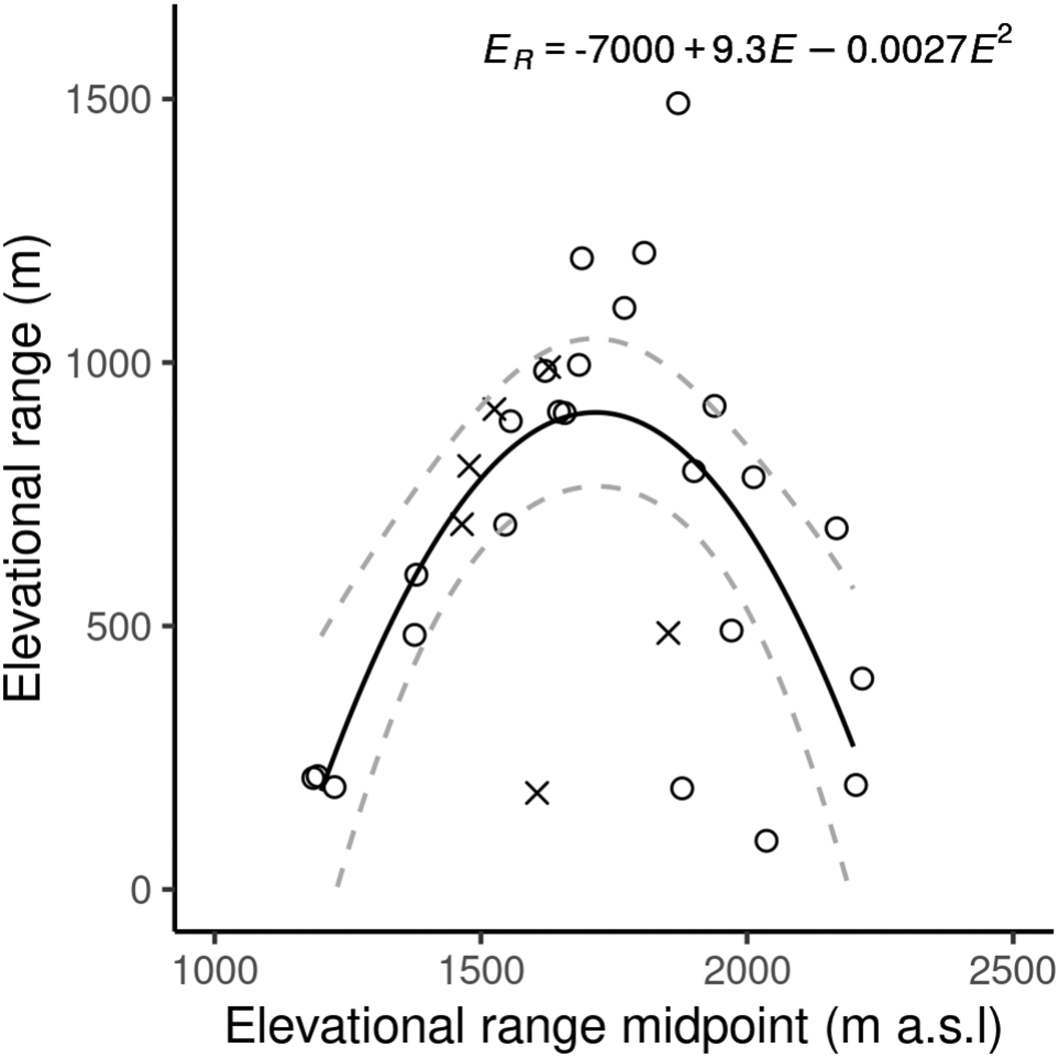
Relationship of elevational range and the elevation range midpoint at which each Orthoptera species was observed, in the Pyrenees. Circles are Caelifera and crosses are Ensifera species. Solid lines show the nonlinear regression for the equation given within the plot and dashed grey lines show the upper and lower bounds of the 95% confidence interval.

Orthoptera recorded to species level and recorded as more than a single individual were used in this part of the analysis (*n* = 609 individuals of 30 species). These 30 species were used to model elevational range against mid‐elevation using nonlinear regressions. The minimum elevational range recorded was 100 m (where species were only found within one elevational band) and the maximum was 1500 m (*Pseudochorthippus parallelus*) which was the only species to be found at both the upper and lower bounds of our sampling (Figure 3). Elevation range showed a hump‐shaped response to elevation with a peak at around 1700 m a.s.l. The quadratic relationship was found to be the best fit (AIC = 428.3, adj *R* ^2^ = .41, *p* < .001; Figure 4) and was significantly better than the linear regression (AIC = 444.0, adj *R* ^2^ = −.03; *F*_1,27_ = 21.38, *p* < .001), so the null hypothesis that there was no difference between the models was rejected. No evidence of the Rapoport effect was found for Orthoptera.

## 4 DISCUSSION

Orthoptera species richness in the Pyrenees mountain range decreased with elevation as predicted by Stevens (1992) (Figure 2), yet contrary to our hypotheses, we found a hump‐shaped response of elevational range (Figure 4) which does not provide support for the Rapoport effect. Elevation was an important predictor of species richness, with sampling effort and vegetation structure providing some influence (Table 1).

We found Orthoptera species richness decreased with elevation (Figure 2). This relationship was consistent with studies of Orthoptera in the French (Claridge & Singhrao, 1978) and Swiss (Descombes et al., 2017; Pitteloud et al., 2020) Alps, dung beetles (Scarabaeinae) in Turkey (Senyuz et al., 2019) and ants in Korea (Kwon et al., 2014). Our results also supported the original prediction made by Stevens (1992) of decreasing species richness along an elevational gradient.

We did not find evidence of the Rapoport effect. Instead, our results showed that Orthoptera at mid‐elevations inhabited a wider range than those at lower and higher elevations (Figure 4). This pattern has previously been found in plants (Bhattarai & Vetaas, 2006; Zhou et al., 2019) but we are not aware of other studies which found peaks of elevational range at mid‐elevations, in other insect taxa. Three out of the four species we found with the highest elevational range midpoint (Figure 3) were species endemic to the Pyrenees or nearby ranges with reported elevations reaching above 2500 m (Poniatowski et al., 2012). Beketov (2009) suggested that the small ranges occupied by these montane specialists would decrease the elevational ranges observed at high elevations and therefore cause a breakdown in the Rapoport effect at this point, which could be a possible explanation for the patterns we see. The reduced wing size of these three endemic species may potentially limit their ability to disperse along the elevational gradient. In contrast, fully‐winged Orthoptera found lower down, are likely to have an increased ability to disperse by flight and therefore may contribute to the peaks of elevational range at mid‐elevations.

Support for the Rapoport effect in insects is varied (McCain & Bracy Knight, 2013; Ribas & Schoereder, 2006). Indeed, we are only aware of three studies which tested directly for, and reported no Rapoport effect for insect taxa (Gaston & Chown, 1999b; Olson, 1994; Shimabukuro & Trivinho‐Strixino, 2021). Contrary to our results, other studies do report an elevational Rapoport effect in invertebrates in Europe (Chatzaki et al., 2005; Rohner et al., 2015), the neotropics (Almeida‐Neto et al., 2006; Brehm et al., 2003; Herzog et al., 2013) and North America (Fleishman et al., 1998; Sanders, 2002). However, given our findings for Orthoptera in the Pyrenees, we do not agree with Herzog et al. (2013) that the Rapoport effect is pervasive across scales. An alternative explanation for smaller ranges at the extremes of our sampling range which led to the mid‐elevation peak is that of Ribas and Schoereder (2006). These authors suggested that observed elevational ranges will be artificially truncated at the upper and lower boundaries of sampling. If species are only recorded over part of their range it follows that their full range has not been observed (Colwell & Hurtt, 1994; Stevens, 1992). This may be an effect acting on the species distributions we observed in our study, particularly at lower elevations. Although it may partly influence the ranges at higher elevations, we think here it is more likely that the presence of montane species or the hard boundaries of mountaintops could be the more important factors driving down the elevational range. For context, studies which did find a Rapoport effect, often, (but not always) sampled over a larger elevational range (e.g. Brehm et al., 2007; Chatzaki et al., 2005; Gaston & Chown, 1999b).

Colwell and Hurtt (1994) and indeed Stevens (1992) in his initial proposal of the Rapoport effect suggested that sampling effort is correlated with the number of species observed, and undersampling of species richness leads to underestimation of elevational ranges. Although sampling varied across sites and we did not record all species present in the region, we accounted for this by including a measure of sampling effort as a confounding variable in our analysis.

Vegetation structure had some influence on the species richness in our study. This has been shown to be an important provider of microclimates and the heterogeneous habitat required for Orthoptera to survive, providing protection from predators and the conditions needed for thermoregulation and reproduction (Cherrill & Brown, 1990, 1992; With et al., 1999). Montane species, which may more easily be able to regulate their temperature (Chappell, 1983), may be more suited to higher elevations where we found less vegetation cover and shorter vegetation. One of our main study areas, Tor, seemed to have been grazed for a longer period and certainly was affected to a greater extent by human activity than La Molinassa and Tavascan, both of which were semi‐natural habitats. Further investigations could focus specifically on the use of habitat by Orthoptera over elevational gradients, to understand these factors.

Orthoptera species distributions are clearly affected by elevation in the Pyrenees but our study did not find a Rapoport effect. The lack of a Rapoport effect is not a novel result for Orthoptera in small regional surveys but it is one of few for insect taxa. Our study points to the importance of understanding the influence of environmental factors on Orthoptera species distributions, and we suggest that conservation efforts in light of climate change will benefit from further studies of these factors.

## AUTHOR CONTRIBUTIONS

**Jen Thomas:** Conceptualization (equal); data curation (lead); formal analysis (lead); investigation (lead); methodology (equal); project administration (lead); software (lead); visualization (lead); writing – original draft (lead); writing – review and editing (lead). **Simon T. Segar:** Formal analysis (supporting); writing – review and editing (supporting). **Andrew J. Cherrill:** Conceptualization (equal); methodology (equal); project administration (supporting); supervision (lead); writing – original draft (supporting); writing – review and editing (supporting).

## CONFLICT OF INTEREST STATEMENT

The authors do not have any competing interests.

### OPEN RESEARCH BADGES

This article has earned Open Data and Open Materials badges.

## Data Availability

Data are available at https://zenodo.org/records/10308140. Code for data recording and management is available at https://zenodo.org/records/7496639 and for the analysis at https://doi.org/10.5281/zenodo.10368290.
